# Incidence and characteristics of bipolar disorder in middle-aged adults: a prospective population-based study

**DOI:** 10.1186/s40345-026-00412-2

**Published:** 2026-01-12

**Authors:** Benjamin Lavigne, Marie-Pierre F. Strippoli, Setareh Ranjbar, Julien Elowe, Sylfa Fassassi, Alexandre Berney, Armin von Gunten, Pierre Vandel, Caroline L. Vandeleur, Martin Preisig

**Affiliations:** 1 Department of Psychiatry, Forensic Psychiatry Unit, Lausanne University Hospital and University of Lausanne, Hôpital de Cery, Route de Cery, 1, Prilly, Switzerland; 2 Department of Psychiatry, Lausanne University Hospital and University of Lausanne, West Sector, Prangins, Switzerland; 3 Psychiatric Epidemiology and Psychopathology Research Center, Lausanne University Hospital and University of Lausanne, Prilly, Switzerland; 4 Department of Psychiatry, General Psychiatry Service, Lausanne University Hospital and University of Lausanne, Prilly, Switzerland; 5 Psychiatric Liaison Service, Department of Psychiatry, Lausanne University Hospital, University of Lausanne, Lausanne, Switzerland; 6 Service of Old-Age Psychiatry, Department of Psychiatry, Lausanne University Hospital and University of Lausanne, Lausanne, Switzerland; 7 Department of Psychiatry, Lausanne University Hospital, Hôpital de Cery, 1 route de Cery, Prilly, Switzerland

**Keywords:** Bipolar disorder, Incidence, Middle-aged adults, Clinical characteristics

## Abstract

**Background:**

Although bipolar disorder (BD) typically emerges in young adulthood, several studies have suggested that the onset of this disorder can occur later in life. However, there are hardly any studies that have established the incidence of BD in older ages and compared clinical features between later-onset and earlier-onset BD. Our study aimed to (1) assess the incidence rate of BD in a population-based prospective study of people older than 35 years, (2) clinically characterize these people with incident BD, and (3) compare their sociodemographic and clinical characteristics with those of people who had already reported lifetime BD at baseline.

**Methods:**

We included 3,709 participants from a population-based cohort study aged 35 to 75 years at the first psychiatric evaluation (mean age 51.4 years, 54.1% women) with at least two psychiatric evaluations. Those exempt from BD at baseline were followed-up (mean duration 11.3 years) to assess the incidence rate of BD. Diagnostic criteria for mental disorders were elicited according to the DSM-IV using the semi-structured Diagnostic Interview for Genetic Studies.

**Results:**

At baseline, 94 participants already met lifetime criteria for BD, whereas five developed BD during the follow-up, corresponding to an incidence rate of 12.2 per 100,000 person-years. Participants who developed BD during the follow-up had a substantially older age at the first episode compared to those who had already reported lifetime BD at the initial psychiatric evaluation (49.8 vs. 29.0 years, respectively). Those with incident BD also reported more frequent initial episodes with mixed symptoms (*p* = 0.003), a shorter duration of initial episodes (*p* = 0.005) and a higher prevalence of pre-existing or co-occurring illicit drug use disorders (*p* = 0.039) than those with pre-existing BD.

**Conclusions:**

Although our results support a later emergence of BD in middle-aged adults, they also suggest atypical first manifestations of this later disorder with a high proportion of mixed episodes and high comorbidity with drug use disorders. From a clinical point of view, our data highlight the necessity for a thorough screening for first manifestations of BD also in middle-aged people particularly in the presence of drug misuse, which may delay the earlier recognition of mood episodes.

## Background

Numerous cross-sectional surveys have provided lifetime prevalence estimates of around 1.0% for bipolar-I disorder (BD-I), 0.8% to 1.6% for bipolar-II disorder (BD-II) and around 1.0% for Other Specified Bipolar and Related Disorders (OSBARD) (Fassassi et al. 2014; Pini et al. 2005; Clemente et al. 2015; Merikangas et al. 2007). In contrast, available data on the incidence rates of these disorders are rare and less accurate. Indeed, establishing the incidence rates of relatively rare disorders including BD is expensive given that it requires that large prospective population-based cohort studies are conducted, which are almost completely lacking. For this reason, available data on the incidence of bipolar disorders (BD) were essentially derived from primary care studies or national patient registers, which however miss untreated cases. Table 1 summarizes studies that have provided incidence rates of BD in six different countries, five of them in Europe, one in Asia. Two relied on data from mental health services (Baldwin et al. 2002; Lloyd et al. 2005), one from physicians in primary care (Kroon et al. 2013) and four from registers in Taiwan (Bih et al. 2008), Denmark (Medici et al. 2015; Jensen and Steinhausen 2016) and Sweden (Söderlund et al. 2015). The two studies conducted in mental health services (Baldwin et al. 2002,Lloyd et al., 2005) used semi-structured diagnostic interviews, whereas the others relied on clinical diagnoses. The studies established incidence rates either for any BD or BD-I, three of them also with sex-specific results (Baldwin et al. 2002; Lloyd et al. 2005; Jensen and Steinhausen 2016). Only the Dutch primary care physician-based study (Kroon et al. 2013) provided data on the incidence of both BD-I and BD-II. The four register-based studies assessed annual incidence rates during the duration of the study. Given the considerable methodological heterogeneity across the seven studies, the incidence of BD overall was found to vary widely, ranging from 4.8 per 100,000 person-years (PY) in the lowest study (Bih et al. 2008) to 28.4 per 100,000 PY in the highest (Medici et al. 2015). The highest rates are in the two studies based on the Psychiatric Central Register of Denmark (Medici et al. 2015; Jensen and Steinhausen 2016). The annual incidence rates provided in the four register-based studies also varied over the years. For BD-I, the estimates established in three studies ranged from 2.2 (Jensen and Steinhausen 2016) to 4.3 per 100,000 PY (Kroon et al. 2013). For BD-II, the Dutch primary care physician-based study (Kroon et al. 2013) provided a lower incidence rate of 1.9 per 100,000 PY (Kroon et al. 2013).

**Table 1:** Review of incidence studies of bipolar disorder since 2002

	Baldwin et al., 2002 (5)	Lloyd et al.,^ 2005^	Bih et al., 2008	Kroon et al., 2013	Medici et al.,^ 2015^	Söderlund et al., 2015	Jensen et al., 2016
**Country**	Ireland	England and Wales	Taiwan	Netherlands	Denmark	Sweden	Denmark
**Recruitment source of cases**	Psychiatric services of the Cavan-Monaghan region and to two major private psychiatric hospitals in Dublin	Mental health services in Nottingham, Lambeth, south-east London, central Bristol	Inpatients and outpatient services of the National Health Insurance program (random sample)	General practice research database containing data from 150 general practitioners	Psychiatric Central Register including psychiatric inpatient and outpatient contacts	National Patient Register including inpatient contacts in Stockholm County	Psychiatric Central Register including all psychiatric inpatient and outpatient contacts
**Study duration**	1995–2000	Nottingham and south-east London: September 1997 - August 1999. Central Bristol: September 1997 - May 1998	1997–2003	1996–2007	January 1995 - December 2012	1955–1967	1995–2010
**Age range**,** years of age**	≥ 15	17–56	≥ 15	≥ 15		18–30	4–65
**Diagnostic assessment**	Structured Clinical Interview for DSM-IV (SCID)	Schedule for Clinical Assessment in Neuropsychiatry (SCAN)	Clinician-based	Clinician-based	Clinician-based	Clinician-based	Clinician-based
**Classification system**	DSM-IV	ICD-10	ICD-9	DSM-IV	ICD-10	ICD-8, ICD-9, ICD-10	ICD-10
**Number of BD cases**	8 BD I	75 BD I	532 BD	70 BD I 31 BD-II	15’334 BD	319 BD	12’034 BD
**Estimated incidence of BD per 100’000 person-years (95%CI)**							
**Any BD**			4.8 – 7.1	7.0 (5.7,8.3)	14.8 – 28.4	7 – 13	12.7 – 21.6
** Women**							14.8 – 26.0
** Men**							11.0 – 17.7
**BD I**	2.2 (0.9,4.3)	4.0 (3.2,5.1)		4.3 (3.4, 5.5)			
** Women**	0.6	4.4 (3.1,6.0)					
** Men**	3.7	3.7 (2.6,5.1)					
**BD II**				1.9 (1.3,2.7)			
**Age at onset of first manic episode**,** years of age (s.d.)**	-	29.2 (9.1)	-	First peak: 15–24 years; Second peak: 45–54 years	-	24.6–26.8 (2.8–4.1)	41.4 (13.1) Men: 43.1 (13.1) Women: 40.8 (13.1)

BD = bipolar disorder, BD-I = bipolar-I disorder, BD-II = bipolar-II disorder, CI = confidence intervals, s.d. = standard deviation

**Table 2:** Description of clinical characteristics of the 5 participants who reported bipolar disorder after the first psychiatric evaluation

Final diagnosis	Sex	Age at first exam	Age at last exam	Age of onset of BD	Type of the initial major mood episode	Duration of initial major mood episode	Functioning during initial major mood episode	Treatment of initial major mood episode	Course after initial major mood episode	Life stress in the year prior to the onset of the initial major mood episode	Lifetime history of psychiatric comorbidity at the time of the initial major mood episode	
BD II	Male	47 years	57 years	54 years	Mixed	3 years	No decrease	Outpatient treatment	Full remission	Marital or professional stressors	None	
BD I	Female	46 years	55 years	48 years	Mixed with psychotic symptoms	8 weeks	Severe decrease	Hospitalization with antipsychotic treatment	Switch to a major depressive episode after 7 months followed by full remission	None	Panic disorder, agoraphobia, and generalized anxiety disorder emerging with the initial mood episode	
BD I	Male	56 years	67 years	56 years	Mixed	14 weeks	Decrease	No	Mixed episode with incomplete remission reverted to a major depressive episode from age 60 to age 64, no treatment, followed by full remission	Bankruptcy of business	Oppositional defiant disorder during adolescence, generalized anxiety disorder emerging with the initial mood episode	
BD II	Male	42 years	56 years	46 years	Mixed	24 weeks	No decrease	Outpatient treatment with antidepressants	Incomplete remission with residual depressive and hypomanic symptoms	None	Alcohol dependence age 22 to 28, cocaine abuse age 22 to 40 follow by cocaine dependence age 40 to 41, specific phobia (fear of heights), emerging with the first mood episode	
BD I	Female	39 years	53 years	45 years	Depressive	20 weeks	Decrease	Outpatient treatment with antidepressants	Full Remission, followed by a 2-year mixed episode at age 50 requiring antidepressant treatment	Job loss	Cannabis abuse since age 17, cocaine dependence age 20 to 35, narcotics dependence age 24 to 36, alcohol dependence since age 45	

BD = bipolar disorder, BD-I = bipolar I disorder, BD-II = bipolar II disorder

**Table 3:** Socio-demographic characteristics according to diagnostic status

	Incident BD after the first psychiatric evaluation	Participants with BP prior to the first psychiatric evaluation	Participants with no BD	Test-statistic	*p*	Pairwise comparison
	*N* = 5	*N* = 94	*N* = 3610			
Female, %	40.0	52.1	54.2	FET	0.751	-
Age at first psychiatric evaluation [years], range, mean (sd)	**45.2 (6.5)**	**49.5 (8.8)**	**51.4 (9.0)**	**F**_**2**_ **= 3.3**	**0.036**	**A**
Age at last psychiatric evaluation [years], range, mean (sd)	57.4 (5.5)	61.2 (8.3)	62.7 (8.9)	F_2_ = 2.2	0.111	-
Socio-economic level^1^, mean (sd)	3.8 (0.4)	3.5 (1.3)	3.4 (1.3)	F_2_ = 0.4	0.666	-

BD = bipolar disorder, sd = standard deviation, FET = Fischer exact test. Significant findings are in bold

Pairwise comparison: A represents the comparison between participants with BD prior to the first psychiatric evaluation vs. participants with no BD

^1^ A value of 3 represents a middle-class socio-economic status on the Hollingshead scale

**Table 4:** Clinical characteristics of participants reporting bipolar disorder prior to and after the first psychiatric evaluation

	Participants with incident BD after the first psychiatric evaluation	Participants with lifetime BD prior to the first psychiatric evaluation	Test- statistic	*p*
	*N* = 5	*N* = 94		
**Initial mood episode**				
Age of onset of BD, mean (SD)	**49.8 (4.9)**	**29.0 (14.0)**	**t=−3.3**	***0.001***
Age range of onset of BD (years)	**39–56**	**6–74**		
Type of episode, % (N)				
Depressive	20.0 (1)	60.6 (57)	FET	*0.157*
Mixed	**80.0 (4)**	**13.8 (13)**	**FET**	***0.003***
Hypomanic	0.0 (0)	14.9 (14)	FET	*1.000*
Manic	0.0 (0)	10.6 (10)	FET	*1.000*
Age of onset of first (hypo)manic episode, mean (SD)	**51.2 (4.3)**	**42.3 (15.7)**	**t=−3.5**	***0.005***
Age of onset of first major depressive episode, mean (SD)	**50.8 (4.6)**	**32.3 (14.5)**	**t=−2.5**	***0.013***
Duration (weeks), median (IQR)	**20.0 (14.0**,**24**,**0)**	**107.9 (38.6**,**260.7)**	**KWT = 4.2**	***0.041***
**Psychiatric disorders preceding or concomitant to the initial mood episode**,** % (N)**				
Any anxiety disorder	60.0 (3)	44.7 (42)	FET	*0.413*
Agoraphobia	20.0 (1)	5.3 (5)	FET	*0.273*
Generalized anxiety disorder	20.0 (1)	3.2 (3)	FET	*0.190*
Panic disorder	20.0 (1)	6.4 (6)	FET	*0.312*
Social phobia	20.0 (1)	19.2 (18)	FET	*0.666*
Specific phobia	20.0 (1)	17.0 (16)	FET	*0.619*
Post-traumatic stress disorder	0.0 (0)	7.5 (7)	FET	*1.000*
Obsessive compulsive disorder	0.0 (0)	6.4 (6)	FET	*1.000*
Any substance use disorder	40.0 (2)	18.1 (17)	FET	*0.244*
Alcohol abuse or dependence	40.0 (2)	14.9 (14)	FET	*0.183*
Illicit drug abuse or dependence	**40.0 (2)**	**5.3 (5)**	**FET**	***0.039***
Behavioral disruptive disorders and Attention-deficit hyperactivity disorder	40.0 (2)	10.6 (10)	FET	*0.110*
Attention-deficit hyperactivity disorder	20.0 (1)	6.4 (6)	FET	*0.312*
Oppositional defiant disorder	20.0 (1)	2.2 (2)	FET	*0.147*
Conduct disorder	0.0 (0)	6.5 (6)	FET	*1.000*
Anti-social personality disorder	0.0 (0)	6.4 6)	FET	*1.000*
**Healthcare use during the most severe mood episode**,** %**				
Consultation with any healthcare professional	60.0 (3)	66.0 (62)	FET	*0.780*
Hospitalization in psychiatric setting	20.0 (1)	16.0 (15)	FET	*0.594*
Psychotropic medication	40.0 (2)	47.9 (45)	FET	*0.786*
Antidepressants	40.0 (2)	31.9 (30)	FET	*0.522*
Mood stabilizers	0.0 (0)	6.4 (6)	FET	*1.000*
Anxiolytics	0.0 (0)	18.1 (17)	FET	*1.000*
Antipsychotics	0.0 (0)	2.1 (2)	FET	*1.000*
**Course characteristics**,** median (IQR)**				
Number of any mood episodes	**3.0 (2.0**,**3.0)**	**5.0 (3.0**,**10.0)**	**KWT = 6.9**	***0.009***
Time spent in mood episodes (weeks)	438.0 (162.0,631.7)	266.8 (130.0,521.4)	KWT = 0.4	*0.533*
Number of (hypo)manic episodes	1.0 (1.0,1.0)	1.0 (1.0,3.0)	KWT = 3.4	*0.066*
Time spent in (hypo)manic episode (weeks)	104.3 (25.7,218.6)	33.2 (8.1,102.7)	KWT = 1.0	*0.326*
Number of major depressive episodes	**2.0 (1.0**,**2.0)**	**3.0 (2.0**,**6.0)**	**KWT = 4.7**	***0.029***
Time spent in major depressive episodes (weeks)	136.3 (39.7,413.1)	185.3 (64.3,402.6)	KWT = 0.2	*0.666*

BD = bipolar disorder, SD = standard deviation, IQR = interquartile range, FET = Fisher’s exact test, KWT = Kruskal-Wallis test, t = Student test. Significant p-values are in bold

Studies focusing on the age of onset of BD have provided highly inconsistent results with mean ages of onset ranging between the twenties and the forties, depending on the age range and the source of investigated samples (general population vs. treated patients). Indeed, within the previously cited research assessing the incidence of BD, two studies reported mean ages of onset in the twenties (Lloyd et al. 2005; Söderlund et al. 2015), whereas the Danish Central Register-based study yielded a mean age of onset slightly higher than 40 years (Jensen and Steinhausen 2016). Pooling data from seven studies together, the review of Goodwin and Jamison (Goodwin and Jamison 2007) showed an age of onset peak in the range of 15 to 19 years, followed closely by the 20 to 24 and 10 to 14 age ranges. The frequency of later ages of onset progressively decreased in older age ranges. In contrast, a recent meta-analysis of 192 epidemiological studies established a median age of onset as high as 33 years for BD (Solmi et al. 2022). Several studies suggested more than one peak of ages of onset. The Dutch general practitioner study provided evidence of two age of onset peaks, one in adolescence and early adulthood and a second one at around 50 years (Kroon et al. 2013). Another clinical study relying on treated patients with BD even suggested three age of onset peaks at the ages of 17.6, 24.6 and 39.2 years (Bellivier et al. 2003). The first peak included 21.4%, the second 57.3% and the third 21.2% of patients. For BD emerging after the age of 50 years, the concept of later-life BD has been developed, which has been postulated to account for approximately a quarter of all cases of BD (Tampi et al. 2021).

Reviews support an association between the age of onset and a series of clinical features (Schürhoff et al. 2000; Weissman et al. 1984; Strober et al. 1988; Joslyn et al. 2016). Early onset was associated with greater severity, higher frequency of comorbid disorders (particularly anxiety and substance use) and poorer long-term outcomes (Schürhoff et al. 2000; Joslyn et al. 2016), whereas the emergence of the disorders in older age was found to be associated with an increased risk of cognitive deficits, physical comorbidities, impaired social functioning and premature death (Shobassy 2021; Beunders et al. 2023). Among the few studies that have compared clinical features of patients by age of onset, a large clinical study conducted in Italy that compared patients with early (age < 18), intermediate (18–40), and later onset (> 40) BD (Miola et al. 2022) found that the latter group of patients had a higher percentage of depressive first episodes, a longer duration of illness, a higher number of depressive episodes, a lower frequency of substance abuse and a higher frequency of physical diseases.

One important limitation of most existing studies on the incidence and age of onset of BD is that they relied on primary care or register-based data and therefore they could not capture persons with less severe forms of BD who do not seek treatment. Moreover, primary care or register-based studies were generally not based on structured or semi-structured diagnostic interviews to systematically assess hypomanic episodes that typically do not entail treatment-seeking. Accordingly, these studies were likely to miss BD-II cases by erroneously assigning a diagnosis of major depressive disorder (MDD), which leaded to an underestimation of the incidence of BD. Similarly, these studies were likely to establish a too late age of onset for BD, if earlier untreated episodes were not assessed. Finally, cross-sectional surveys in the community were likely to yield inaccurate estimates of the onset of disorders due to an incomplete or inaccurate recall of milder episodes or of the onset of the first episode.

Using data from a prospective population-based cohort study including people aged 35 years and older, the goals of the present paper were to (1) determine the incidence of BD during the follow-up of this cohort in people exempt from this disorder at baseline, (2) clinically characterize these incident BD cases in terms of the type and duration of the initial mood episode, the presence of psychiatric comorbidity, health care use during the initial episode and the course of the disorder following the initial episode, and (3) compare people with incident BD during the follow-up to those who already had a lifetime history of BD at baseline with respect to sociodemographic and clinical course characteristics including age, type and duration of the initial mood episode, psychiatric comorbidity, and health care use at the time of the most severe episode.

## Methods

### Study sample

The present data stemmed from CoLaus|PsyCoLaus, a longitudinal population-based study designed to prospectively assess the associations between mental disorders and cardiovascular risk factors in the community. The methodological features of this study were previously described in detail (Firmann et al. 2008; Preisig et al. 2009). Briefly, CoLaus|PsyCoLaus initially included a random sample of 6,734 participants (age range: 35–75 years) selected from the civil register of the city of Lausanne (Switzerland) between 2003 and 2007. The sample underwent a comprehensive investigation of cardiovascular risk factors and was also invited to participate in a psychiatric evaluation. Physical and psychiatric followed-up investigations were completed after approximately 5 (follow-up 1, FU1), 9 (follow-up 2, FU2) and 13 years (follow-up 3, FU3). The present analyses included participants with at least two psychiatric evaluations (*n* = 3,758). Among them, participants with a lifetime history of schizophrenia (*n* = 10), schizophreniform disorder (*n* = 1) or schizoaffective disorder (*n* = 38) at the first psychiatric evaluation were excluded, resulting in a final sample of 3,709 participants.

### Assessments

Diagnostic information was collected using the semi-structured Diagnostic Interview for Genetic Studies (DIGS) (Nurnberger et al. 1994). The DIGS was originally designed to elicit information on a wide spectrum of DSM-IV Axis-I criteria as well as on healthcare use and symptoms for all assessed disorders. The French version of the DIGS revealed excellent inter-rater and fair to good test–retest reliability for major mood (Preisig et al. 1999) and substance use disorders (SUD) (Berney et al. 2002). The DIGS was completed with anxiety disorder sections of the French version (Leboyer et al. 1991) of the Schedule for Affective Disorders and Schizophrenia—lifetime and anxiety disorder version (SADS-LA) (Endicott and Spitzer 1978). At the follow-up evaluations, a shortened version of the DIGS was used to assess the occurrence of psychopathology since the last assessment. Medical records were also systematically collected and diagnostic information elicited by the DIGS interviews and the medical records were combined. Diagnoses at baseline were assigned according to the diagnostic lifetime information collected at baseline, whereas lifetime diagnoses at follow-up were assigned combining the cumulative diagnostic information until the last psychiatric evaluation. Diagnoses of mood disorders were assigned according to the DSM-5. Manic or hypomanic episodes with mixed features (presence of at least 3 out of 6 specified depression symptoms according to the DSM-5) and major depressive episodes with mixed features (presence of at least 3 of 7 specified manic/hypomanic symptoms according to the DSM-5) were considered as mixed episodes. Diagnoses of non-mood disorders were assigned according to the DSM-IV. The DIGS also collects sociodemographic information and contains questions on health care and drug use. The level of socio-economic status (SES) was determined using the Hollingshead scale (Hollingshead 1975), which takes into account the highest level of education and professional occupation attained. Interviewers were master’s-level psychologists, who were trained over a one- to two-month period. An experienced senior psychologist reviewed all interviews. In addition to the DIGS, information from medical records was used where available.

### Ethics approval and consent to participate

The institutional Ethics Committee of the University of Lausanne, which afterwards became the Ethics Commission of the Canton of Vaud (www.cer-vd.ch) approved the baseline CoLaus|PsyColaus study (reference 16/03; 134-03,134-05bis, 134-05-2to5 addenda 1to4). The approval was renewed for the first (reference 33/09;239/09), the second (reference 26/14; 239/09 addendum 2) and the third (PB_2018-00040; 239/09 addenda 3to4) follow-ups. The study was performed in agreement with the Helsinki declaration and its former amendments, and in accordance with the applicable Swiss legislation. All participants signed a written informed consent.

### Statistical analyses

The calculation of the incidence rate of BD indicated per 100,000 (PY) relied on the sample of participants who were exempt from lifetime BD at baseline. The rate was calculated as the number of new BD cases between the first and last psychiatric assessments, divided by the sum of the observation periods of all participants until either the date of the onset of BD or the date of the last psychiatric evaluation. Sociodemographic characteristics were compared across groups of participants with incident BD during the follow-up, those with a lifetime history of BD at the first psychiatric evaluation and those with no BD using Fisher exact tests, and ANOVA where appropriate. The lifetime history of anxiety, SUD, behavioral disruptive disorders, attention-deficit hyperactivity disorder (ADHD) and antisocial personality disorder as well as health care use during the most severe mood episode and the age of onset, the type and duration of mood episodes were compared across groups of participants with incident BD during the follow-up, and those with a lifetime history of BD at the first psychiatric evaluation using Fisher exact tests, Student tests and Kruskal-Wallis tests where appropriate. Statistical analyses were conducted using the Statistical Analysis System, version 9.4 for Windows (SAS Institute Inc., Cary, NC, USA).

## Results

### Description of the cohort and incidence rate

At the first psychiatric evaluation, the mean age of the 3,709 participants who could be included in the follow-up was 51.4 years (s.d: 9.0 years; range: 35–76 years). The proportion of women was 54.1% and the mean SES was 3.4 (s.d. 1.3) according to the Hollingshead scale (range 1–5, with higher values indicating high SES, 3 reflecting middle-class SES). At the first psychiatric evaluation, already 61 participants reported a lifetime history of BD. In addition, among those who already had a lifetime diagnosis of MDD at the first psychiatric evaluation, there were 33 who developed a first manic or hypomanic episode during the follow-up. Because their initial depressive episode retrospectively marked the onset of BD, these participants were reclassified as already affected by BD at baseline rather than considered incident cases. This increased the number of participants with BD at the first psychiatric investigation to a total of 94 and reduced the number of participants exempt from BD at baseline to 3,615. These participants were followed up across a mean duration of 11.3 (s.d. 3.5) years. At the end of the follow-up, their mean age was 62.7 (s.d. 8.9) years. Among them, five developed a first episode of BD. The number of PYs during the follow-up period was 40’900, resulting in an estimate of the incidence rate of 12.2 (95% confidence interval: 6.4–21.2) per 100,000 PY.

### Clinical characteristics of participants with incident BD during the follow-up

The clinical characteristics of the five participants with incident BD are provided in Table 2. Three of them met criteria for BD-I. Two of the five participants were women. At the first psychiatric investigation, the five participants’ ages ranged between 39 and 56, at the last psychiatric investigation between 53 and 67. The onset of their first major mood episode lay between ages 45 and 56 years. Four participants started with a mixed episode and one with a major depressive episode (MDE). Among the four participants who started with a mixed episode according to the DSM-5, two would have also fulfilled the more stringent DSM-IV criteria (simultaneously meeting full criteria for a manic and a depressive episode during one week). One of those with a mixed episode also experienced psychotic symptoms. The initial mood episodes lasted between 8 weeks and 3 years. In three participants the initial mood episode was accompanied by a decrease in functioning. One participant received inpatient treatment, three outpatient treatment, and one participant was not treated. The initial episode fully remitted in two participants, whereas two participants reported partial remission with residual symptoms and one participant switched to a MDE followed by full remission. Three of the five participants with incident BD during the follow-up reported significant life stressors during the year prior to the first mood episode. Four participants with incident BD also reported multiple psychiatric non-mood disorders that preceded the onset of the first major mood episode or jointly emerged with this episode. Among them, two had a lifetime history of anxiety disorders other than specific phobia, one of them also reported oppositional defiant disorder. Two other participants had SUD combining alcohol and illicit drug dependence. One of them also reported an earlier minor depressive episode.

### Comparisons across diagnostic groups

Table 3 provides the sociodemographic characteristics of the five participants with BD after the baseline assessment, the 94 participants with onset of BD prior to the first psychiatric evaluation and the 3610 participants without BD. The only intergroup difference was that participants with prior BD were younger than those with no BD at the first psychiatric evaluation. The comparison of clinical characteristics between participants with BD emerging before and after the first psychiatric evaluation is provided in Table 4. The mean age of onset of the initial mood episode of the five participants with incident BD lay at almost 50 years compared to 29 years in those with a BD that emerged prior to the first psychiatric evaluation. Regarding the polarity of the initial mood episode, four out of the five participants with incident BD started with a mixed episode. This proportion was significantly higher than that of 13.8% of the participants with BD at baseline, whereas among the participants of the latter group, more than six out of ten started with a MDE. Participants with incident BD had a higher age of onset of the first (hypo)manic and the first depressive episodes. With a duration of 20 weeks, the initial mood episode in BD emerging during the follow-up was much shorter than that of the more than 100 weeks that occurred prior to the first psychiatric evaluation. Regarding psychiatric non-mood disorders that occurred earlier or simultaneously to the onset of BD, participants with incident BD differed from those with BD at baseline with respect to illicit drug use disorders. Indeed, the proportion of two out of the five participants in this group reporting this disorder was significantly higher than that of the 5.3% in the other participants with BD. However, participants with incident BD did not differ from participants with BD at baseline in health care use during the most severe episode. With respect to course characteristics, participants with incident BD reported a lower number of any mood episodes and MDE than those with BD at the first psychiatric evaluation.

## Discussion

To our knowledge, this is one of the very few prospective, population-based studies focusing specifically on middle-aged adults. Based on a unique dataset from a prospective cohort study that involved repetitive assessments of 35 to 75-year-old participants from the general population, using semi-structured diagnostic interviews over 11 years, the most salient findings were that: (1) we could identify five participants exempt from BD at the first psychiatric evaluation who developed BD during follow-up, corresponding to an incidence rate of 12.2 per 100,000 PY, and (2) aside from the much higher age of onset and, as a likely consequence, a smaller total number of episodes, these five participants with incident BD were characterized by a more frequent onset of the disorder with a mixed episode, a shorter duration of the initial mood episode, and more frequent illicit drug use disorders as compared to participants with BD already reported at the first psychiatric evaluation.

### Incidence of BD

Hence, our results support the emergence of new BD after the age of 35 years, i.e. the age of the inclusion of the youngest participants in our study. The established incidence of 12.2 per 100,000 PY in our middle-aged cohort cannot be easily compared with estimates from previous research, given that we are not aware of previous studies that specifically assessed the incidence of BD in middle-aged people. Our incidence rate lay within the ranges reported by the Danish and Swedish register-based studies (Medici et al. 2015; Jensen and Steinhausen 2016; Söderlund et al. 2015), although these studies also included younger age-ranges with a potentially higher incidence of BD. In contrast, these studies could not identify non-treated mood episodes and may therefore have missed BD-II in particular.

### Clinical characteristics

Given the low number of only five incident cases of BD, our analyses comparing these five participants with those who had already reported BD at baseline involved low statistical power and we could only apply the Fisher’s Exact test, which cannot adjust for other variables. Hence, our results suggesting that the five incident cases significantly differed from those with prior BD with respect to three clinical features should be interpreted with caution. The most remarkable clinical difference was that four out of the five participants with incident BD started with a mixed episode according to the DSM-5 and only one with a MDE. If we had applied the more stringent DSM-IV criteria for mixed episode still two out of these four participants would have started with a mixed episode, i.e. 40% of the five incident cases. Among participants with onset of BD prior to the first psychiatric evaluation, more than 60% started with MDE, which is consistent with the bulk of existing literature essentially based on clinical samples showing that more than 50% of patients with BD start with depressive episodes (Baldessarini et al. 2014; García-Jiménez et al. 2020; Brancati et al. 2023; Wang et al. 2021). In contrast, onsets with mixed episodes generally based on DSM-IV criteria were reported in less than 10% of patients with BD (Baldessarini et al. 2014; Brancati et al. 2023).

With a median duration of 20 weeks, the first episodes in the five participants with incident BD in our study were shorter than the more than two-year median first episode duration of the participants reporting BD already at the first evaluation. The 20-week duration of the first episode of incident BD in our study lies within the range of one to six months established for the duration of mood episodes in previous studies on patients with BD, although this research rarely documented the duration of first episodes (Tondo et al. 2017; Solomon et al. 2010). However, the median duration of more than two years of the first episode of participants who reported already a BD at the first evaluation is surprisingly long. Such long episodes with durations up to two years have been previously only described in BD with different forms of rapid cycling (Tondo et al. 2017). Given that the initial episode of participants who reported BD already at their first evaluation was generally a MDE with no indication of mixed features or rapid cycling, we have to assume that the unusual length of the first episode in these participants is attributable to inaccurate recall. Indeed, episodes with incomplete remission or recurrent episodes with short inter-episode intervals may have led to the reporting of such long episode durations. The risk of such information bias was higher in participants with BD at baseline given the potentially longer interval between the onset of the BD and the first psychiatric evaluation in these participants as compared to those with incident BD who had their first mood episode within a maximum five-year follow-up interval.

Another clinical characteristic that significantly differed between incident BD and BD already reported at baseline was the high proportion of participants with pre-existing or co-occurring illicit drug use disorders. This finding contrasts with studies suggesting that SUD are more frequently associated with early-onset BD (Strakowski et al. 1996; Hunt et al. 2016). However, it is possible that the combined alcohol and illicit drug use disorders in two out of the five participants with incident BD may have masked earlier mood episodes and thereby led to a differed detection of the onset of BD. However, it is also possible that the SUD in these participants triggered the later onset of BD in people with lower vulnerability to BD. In contrast to previous research, the participants with incident BD, who had a much higher age of onset, did not report a lower prevalence of comorbid psychiatric disorders or indicators for lower disorder severity as compared to those with BD at baseline (Shobassy 2021; Beunders et al. 2023).

Finally, potential hormonal influences should also be considered in the interpretation of our findings. The two women who converted had their first (hypo)manic episodes at the ages of 48 and 45 years. Given these ages, it is possible that features associated with pre-menopause or menopause contributed to the development of these first (hypo)manic episodes.

### Limitations

The main limitation of our study is the small size of the cohort for identifying rare events such as the incidence of BD during the follow-up period, which is reflected by the large width of the confidence interval of the established incidence rate. The small number of cases with incident BD during the follow-up also entailed restricted power to detect sociodemographic or clinical differences between incident BD and BD already reported at the first psychiatric investigation and only Fisher exact tests could be conducted, which did not allow us to adjust for potential confounding.

Another limitation was the relatively high age of participants at baseline (35–75 years). Participants may have failed to recall distant mood episodes or may have only partially remembered their symptoms. Such recall bias could have led to an overestimation of incident-BD cases during the follow-up if episodes that had occurred before the first psychiatric evaluation were not reported. In particular, one participant who reported a mild depressive episode prior to the first evaluation may have not recalled all earlier potential symptoms.

## Conclusions

Our results support the occurrence of BD onset after the age of 35 years even if this is a rare event, with an incident rate within the usual range documented in the literature for BD. Although the number of the participants with incident BD is low, and therefore our results should be interpreted with caution, they also suggest atypical first manifestations of the disorder, with a high proportion of mixed episodes and high comorbidity with illicit drug use disorders. Some of these disorders could have emerged earlier in life, where the drug use disorders could have masked the detection of earlier mood episodes. Ideally, our findings should be replicated in long-term prospective studies exclusively including adolescents or young adults at baseline, thereby minimizing the risk of unrecalled first mood episodes. From a clinical point of view, our data highlight the necessity for a thorough screening for first manifestations of BD also in middle-aged people, particularly in the presence of illicit drug use disorders which may have delayed the earlier recognition of mood episodes.

## Data Availability

The data of CoLaus|PsyCoLaus study used in this article cannot be fully shared as they contain potentially sensitive personal information on participants. According to the Ethics Committee for Research of the Canton of Vaud, sharing these data would be a violation of the Swiss legislation with respect to privacy protection. However, coded individual-level data that do not allow researchers to identify participants are available upon request to researchers who meet the criteria for data sharing of the CoLaus|PsyCoLaus Datacenter (CHUV, Lausanne, Switzerland). Any researcher affiliated to a public or private research institution who complies with the CoLaus|PsyCoLaus standards can submit a research application to research.colaus@chuv.ch or research.psycolaus@chuv.ch. Proposals requiring baseline data only, will be evaluated by the baseline (local) Scientific Committee (SC) of the CoLaus and PsyCoLaus studies. Proposals requiring follow-up data will be evaluated by the follow-up (multicentric) SC of the CoLaus|PsyCoLaus cohort study. Detailed instructions for gaining access to the CoLaus|PsyCoLaus data used in this study are available at [www.colaus-psycolaus.ch/professionals/how-to-collaborate/].

## Abbreviations

ADHD: Attention-deficit hyperactivity disorder
BD: Dipolar disorder
BD-I: Bipolar-I disorder
BD-II: Bipolar-II disorder
DIGS: Diagnostic interview for genetic studies
FU: Follow-up
MDD: Major depressive disorder
MDE: Major depressive episode
OSBARD: Other specified bipolar and related disorders
PY: Person-years
SADS-LA: Schedule for affective disorders and schizophrenia—lifetime and anxiety disorder version
SES: Socio-economic status
SUD: Substance use disorders
